# Spatial heterogeneity in repeated measures of perceived stress among car commuters in Scania, Sweden

**DOI:** 10.1186/s12942-016-0054-8

**Published:** 2016-07-27

**Authors:** Kristoffer Mattisson, Kristina Jakobsson, Carita Håkansson, Ellen Cromley

**Affiliations:** 1 Department of Occupational and Environmental Medicine, Lund University, 221 85 Lund, Sweden; 2 Department of Community Medicine and Health Care, University of Connecticut School of Medicine, 263 Farmington Avenue, MC 6325, Farmington, CT 06030-6325 USA

**Keywords:** Stress, Commuting, Cross-sectional, Repeated measures, Spatial heterogeneity, Geographically weighted proportions, Sweden

## Abstract

**Background:**

Long commutes by car are stressful. Most research studying health effects of commuting have summarized cross-sectional data for large regions. This study investigated whether the levels of stress and individual characteristics among 30–60 min car commuters were similar across different places within the county of Scania, Sweden, and if there were changes over time.

**Methods:**

The study population was drawn from a public health survey conducted in 2000, with follow-ups in 2005 and 2010. The study population was selected from the 8206 study participants that completed the questionnaire at all three time points. Commuting questions in the 2010 questionnaire assessed exposure concurrently for that year and retrospectively for 2000 and 2005. In total, 997 persons aged 18–65 and working 15–60 h/week had commuted by car 30–60 min at least at one time point. Geographically weighted proportions of stress among 30–60 min car commuters were calculated for each year and classified into geographically continuous groups based on Wards algorithm. Stress levels, sociodemographic characteristics and commuting characteristics were compared for areas with high and low stress in relation to the rest of the county. This novel methodology can be adapted to other study settings where individual-level data are available over time.

**Results:**

Spatial heterogeneity in stress levels was observed and the locations of high and low stress areas changed over time. Local differences in stress among participants were only partly explained by sociodemographic characteristics. Stressed commuters in the high stress area in 2000 were more likely to maintain their commuting mode and time than those not stressed. Stressed commuters in the high stress area in 2000 were also more likely to have the same workplace location in 2010, while stressed commuters in the high stress area in 2010 were more likely to have the same residential location as in 2000.

**Conclusion:**

The relationship between commuting mode and time and stress is variable in place and time. Better understanding of commuting contexts such as congestion is needed in research on the health effects of commuting.

## Background

Commuting, the regular travel between home and work, accounts for a significant block of time in everyday life. The distance and time associated with the journey to work has been increasing in many regions [1, 2]. According to economic theory, regions benefit from larger labor markets and individual commuters are compensated by access to better jobs, higher salaries, lower rents or greater choice of residential locality. Some empirical studies report that people who spend more time commuting have lower subjective well-being, suggesting a “commuting paradox” in which the cost of longer commutes is not offset by advantages in other aspects of life [3].

In health research, commuting has been shown to be associated with stress [4–6]. Commuting makes recurrent and enduring demands on individuals which require them to readjust their behaviors over long periods of time to cope [7]. Subjective and objective stressors such as crowding, lack of control and flexibility, and noise associated with the commute itself may give rise to subjective responses [4]. The perceived stress can directly, and via triggered physiological responses, have negative impacts on attitudes, behavior and health. Increased stress may also occur as a function of the time lost while commuting [8]. More time spent on the journey to work means less spare time for health beneficial activities and can cause disturbances in work–family balance. Thus, stress experienced due to the commute contributes to the everyday stress experienced at the workplace and at home [9].

Stress in commuting is likely a combination of individual factors, and characteristics of the commute itself such as journey duration [5, 10]. Environmental exposure such as congestion, perception of the surroundings, noise, and air pollution is believed to cause stress among commuters [4]. These environmental factors vary for different places. The association between commuting and stress could therefore also vary spatially, depending on the context where the commuting takes place.

There is increasing interest in understanding spatial heterogeneity in patterns of commuting [11]. A number of studies focus on forms of active commuting. In Paris, a study of individual and environmental factors and active commuting found differences from place to place in the relationship between some of the environmental factors and walking or cycling to work [12]. In a Dutch study, geographic heterogeneity in cycling under different weather conditions was considered [13]. An ecological study in the US considered how environmental factors influence the use of active transportation [14]. There has been surprisingly little study connecting commuting to the residential locations of commuters [15]. In a Swedish study, commuters who traveled a long duration (mode was not known) and resided in metropolitan areas experienced higher separation rates from their partners compared to commuters living in rural areas after adjustment for individual characteristics [16].

This study focuses on spatial variability in stress among 30–60 min car commuters in the county of Scania, Sweden. A cross-sectional study in the county comparing active (walking or cycling), car, and public transit commuters found a stronger association between car and public transit commuting and high stress than with active commuting [6]. The strongest association with high stress was found among 30–60 min car commuters. Studies conducted in other countries in Europe and North America and in Australia have also shown that car commuting is perceived as more stressful than public transit or active commuting [17–21].

Commuting time and distance have been increasing in Scania [22] and in the rest of Sweden [23], and commuting by car is the main mode [24]. A large pool of car commuters presently commutes close to 30 min one way. Continuing increases in commuting distance will likely lift a lot of commuters to the 30–60 min category, which would pose a potential problem of higher stress for a larger number of commuters. The patterns of commuting in Scania are similar to those in other areas of Sweden and countries in Europe and North America in terms of reliance on the car and commuting times [3, 25, 26]. This creates an opportunity to assess geographical variability in the association between a common form of commuting and a range of health effects.

Much of the research on health effects of commuting to date has not explicitly addressed whether these effects are more pronounced in some areas than others. Research conducted in some communities finds evidence of health effects [4–6] while research conducted in other settings suggests no association between commuting and a range of health outcomes [27]. Spatial analyses in health provide insight into variations in the associations between commuting and health within and across countries. The novel methodology used in this analysis can be easily adapted to other study settings.

The general aim of the paper was to study whether the level of stress among 30–60 min car commuters in Scania was similar across space or if it differed between different places and whether differences across places varied over time. A secondary aim was to study whether these potential differences in stress among 30–60 min car commuters in place and time were accompanied by differences in socio-demographic characteristics of commuters or the spatial contexts of their commutes. The final aim of the research was to investigate whether the potential changes in the location of the high stress areas over time could be explained by migration in and out of the areas, change of workplaces, or adoption of other commuting modes as ways of dealing with a stressful commute. We hypothesized the existence of geographic disparities in the association between a 30–60 min car commute with self-reported stress over time within Scania. We expected that 30–60 min car commuters in areas where such commuters reported higher stress would have distinctive socio-demographic characteristics, such as being a woman, having a low level of education, low income or low occupational status [28, 29], associated with higher stress levels. Finally, we expected that changes in the spatial location of high stress areas could be associated with high levels of mitigation, with individuals in high stress areas changing workplaces and commuting modes.

## Methods

### Study area

**Fig. 1:**
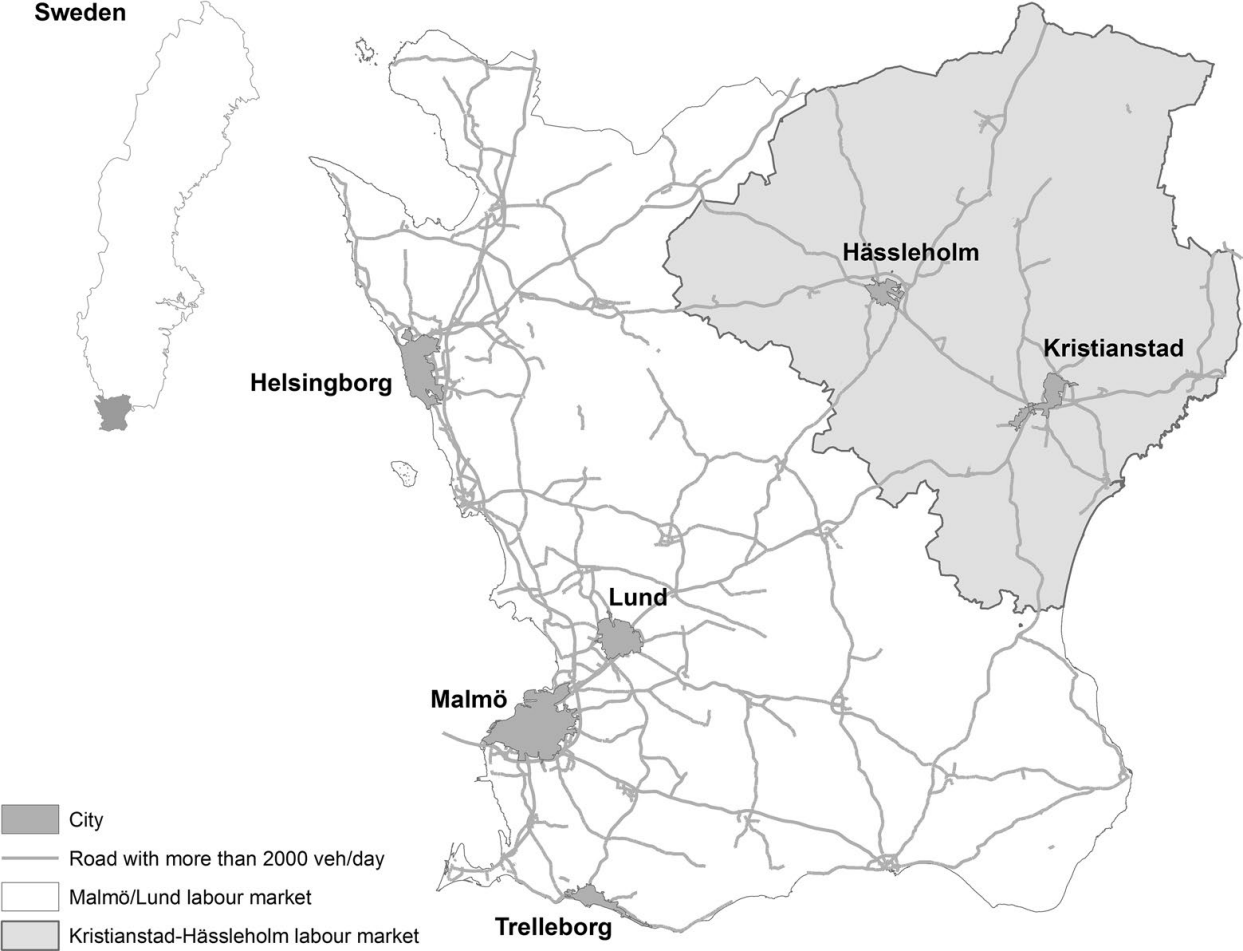
The study area

Scania is the southernmost county of Sweden (Fig. 1). It is characterized by a polycentric city structure, where people often live in one place and work in another [30]. Scania covers almost 11,000 km^2^ and has a population density of 116 inhabitants/km^2^. The county has seven regional cores but Malmö, Lund and Helsingborg are the strongest contributors to population and employment growth [30]. In 2000, the Öresund bridge was opened connecting Malmö and the Danish capital Copenhagen, creating the core Öresund region, the largest and most densely populated metropolitan area in the Nordic countries with 3.7 million inhabitants. Infrastructure is well developed to the west, and future investments are planned to increase connections between west and east.

### Study population

The study population was selected from repeated cross-sections drawn from participants in a public health survey, Public Health in Scania (PHS), sent to 24,922 persons in 2000. The selection procedure at baseline was geographically stratified based on the 33 municipalities in Scania. Strata were also formed within each municipality based on gender. Two follow-ups were performed in 2005 and 2010 [31]. In 2000, 13,604 (55 %) persons responded. Study subjects were selected from the 8206 respondents who answered the questionnaire at all three time points. The questionnaire contained more than one hundred questions about background, family situation, work and health. The questionnaire in 2005 was identical to the one from 2000. In 2010, eight additional questions were added. Questions about commuting duration and mode were added to PHS in 2010, including self-report of commuting mode and time in 2010 and retrospective self-report of commuting mode and time for 2000 and 2005.

All of the 8206 respondents who fulfilled the inclusion criteria were eligible for this study. The inclusion criteria were that the commuter should be between 18 and 65 years old in a given year, commuted 30–60 min by car, worked 15–60 h/week, answered the public health survey question about stress, and had residential coordinates linked from register data. Based on these inclusion criteria, we included a total of 997 individuals of which 616 were eligible in 2000, 543 in 2005 and 454 in 2010.

### Register data

Register data on income, occupational status and workplace location were obtained from Statistics Sweden (SCB). Workplace location was provided for the northwest coordinates of the grid cell location of the workplace. Grid cell size was 250 × 250 m within cities and 1000 × 1000 m outside cities. Residential locations were obtained from regional authorities based on the centroid of the real estate parcel where the study subject lived. All locations were given in Swedish grid system coordinates (RT 90 2.5gon west) and were projected not geographic coordinates. Ethical approval was granted by the regional ethical review board in Lund to connect the data from SCB to the survey data and to conduct the study.

### Health outcome

Stress was measured as perceived stress based on one question in the survey: “Do you feel stressed in your everyday life?” with three response alternatives (1) Yes, in general (2) Yes, sometimes (3) No (almost never). This variable was dichotomized into stressed (1) and not stressed (2–3). This strict definition was used to include only those strongly perceiving stress.

### Sociodemographic characteristics of commuters

*Age* was obtained from the questionnaire as self-reported for each year. This information was dichotomized into 50 years or older and younger than 50 years. This variable was included to represent two different stages in life. Research has shown that car ownership tends to increase to the age of fifty and thereafter decline [32].

*Gender* was obtained from the questionnaire. Women often have greater responsibilities for taking care of the home and family, which could be expected to have an influence on the stress experienced in relation to commuting [33]. The degree of compensation for employment requiring longer commutes might also differ by gender [34].

*Educational level* was acquired from the questionnaire for each year. The question included ten levels from completing primary school (1–6 years) to research education. This question was dichotomized into more than 12 years of school and less than or equal to 12 years of school. Educational status is a socioeconomic measure that could indicate income, family background and motivation [35].

*Occupational status* was obtained from Statistics Sweden in six categories: unskilled and skilled manual workers, non-manual employees on a low, medium and high level and “farmers and entrepreneurs”. These were dichotomized into blue collar occupations (unskilled and skilled manual workers; including also farmers and entrepreneurs) and white collar occupations (non-manual employees on a low, medium and high level). Different types of job could generate different levels of stress [36].

*Job satisfaction* was measured with the question “Is the company/workplace that you are working at today the one that you wish to work for in the future?” with the alternatives yes or no. Job satisfaction could compensate the negative stress experienced by the commute [37].

*Income* was obtained from Statistics Sweden as the disposable income of the individual, and adjusted for inflation. A higher income would suggest a greater compensation for the commute [38].

*Living alone* was self-reported based on the question “How many persons, including yourself, are living in your household?”. All answering “1” were classified as living alone. Living alone would mean not having a family at home to consider and would thereby minimize work–family conflict [9].

*Rooted in neighborhood* was self-reported based on the question “Do you feel rooted and have a strong sense of belonging with your residential area?” (1) to a high degree (2) to some extent (3) not especially (4) not at all. This question was dichotomized into: high neighborhood connection (1) and low neighborhood connection (2–4). We use neighborhood connection as an indicator of residential satisfaction, thus compensating for commuting [3].

### Commuting characteristics

*Mode and time* Car commuters traveling 30–60 min one way were identified based on the self-reported questions: “How much time does it take to get to work (single journey)?” (1) <15 min (2) 15–30 min (3) 30–60 min (4) 1–1.5 h (5) 1.5–2 h and (6) longer than 2 h and a multiple choice question “How do you usually go to work?” (1) walking (2) cycling (3) car (4) bus (5) train (6) other (7) do not work. Participants answering 30–60 min commute and car (only car or car with walking or cycling on the multiple choice question) were coded as 30–60 min car commuters. This approach to classify commuters by mode and time has been used in other studies relying on public health survey data in Sweden [1, 6].

*Distance* Euclidean distance between residence and workplace was calculated based on projected coordinates of the residential location and of the workplace location. Distance in addition to mode and duration is an important characteristic of the commute. A 30-min commute in a congested area might cover a much shorter distance than a 30-min commute in a rural area with less traffic. This variable was also dichotomized into commuting longer or shorter than the median distance for all 30–60 min car commuters that year.

*Living or working in a big city* was measured as residence or workplace located within the city borders of Malmö or Lund. Malmö and Lund are the largest cities in the region and commuting into or out of this area would be associated with more traffic and congestion.

### Analysis

The analysis proceeded in three stages. First, we conducted a spatial analysis using geographically weighted proportions to assess the proportion of stress among neighboring commuters of each participant for each year. A geographically weighted proportion (GWP) is a spatial statistical measure [39] which was calculated based on the residential location of each participant using Gaussian spatial weights and a fixed bandwidth of 20 km. The bandwidth was selected to define the local area within which commuters would experience similar residential, employment, and commuting opportunities. This distance defines a zone around each residential location that is similar in size to the administrative *kommuner* or county sub-units in Scania. The GWP was calculated using the formula given by Fotheringham et al. [40].

After calculating the GWP for each participant residential location in a particular year, Ward’s classification analysis was applied to the geographically weighted proportions to group all study subjects for that year. Geographically contiguous areas with at least 30 participants who had similar GWP values were identified.

The proportion of 30–60 min car commuters who reported stress was calculated for participants in each group. Areas with the highest and the lowest proportions of stress for each year were selected for further analysis.

In the second stage, we compared the sociodemographic and commuting characteristics of the individuals in the areas with the highest and lowest proportion of stress with 30–60 min car commuters in the county as a whole for each of the 3 years separately. Differences in local area proportions were calculated for covariates describing sociodemographic and commuting characteristics among commuters in the high and low stress areas and the remaining study population. Z-scores were calculated based on the formula for a test of single proportions [41]. The null hypothesis was rejected if the Z-score exceeded the critical value for a two-tailed test. If the null hypothesis was rejected, there was a difference in the sociodemographic or commuting characteristic in the high or low stress area.

The final step in the analysis was to investigate how reported stress in the high stress areas in 2000 and 2010 were affected by changes in residential location and commuting among study subjects over time. That is, stress in the highest stress area in 2000 might have decreased because commuters experiencing stress in 2000 changed their residential locations or modes of commuting by 2010. For all residents of the high stress area in 2000, the proportions of commuters who by 2010 had changed residential location, workplace location, and commuting were assessed. For all residents of the high stress area in 2010, the proportion of commuters who since 2000 had changed residential location, workplace location, and commuting were also assessed.

The stress levels of commuters moving within and out of the 2000 and within or into the 2010 high stress areas were also compared. Changes in the observed levels of self-reported general stress in an area could result from change in the local population or from more- or less-stressed individuals moving into or out of the area.

## Results

### Spatial heterogeneity

**Fig. 2:**
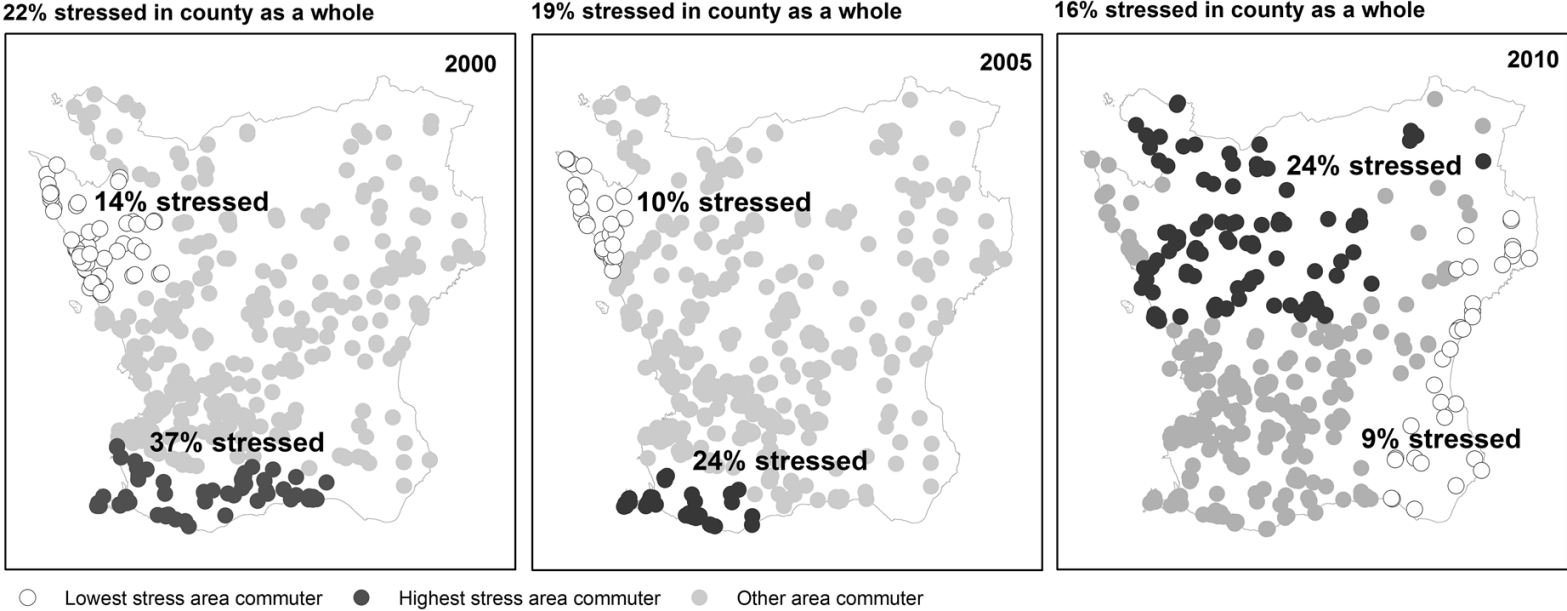
Areas with the highest and lowest proportions of stressed 30–60 min car commuters by year with proportion stressed among 30–60 min car commuters in the county as a whole

Grouping observations based on geographically weighted proportions of stress among 30–60 min car commuters indicated spatial heterogeneity in the levels of self-reported stress among 30–60 min car commuters in each year (Fig. 2). In 2000, the overall proportion of stress in 30–60 min car commuters in the county was 22 %. In the area with the highest stress level based on analysis of GWPs, the simple proportion of 30–60 min car commuters reporting stress was 37 %. In the area with the lowest stress level, the simple proportion of such commuters reporting stress was only 14 %.

The overall stress level among 30–60 min car commuters decreased from 2000 to 2010, but geographical differences in the levels of stress were apparent across the 3 years. The highest and lowest stress areas were located in the same parts of the county in 2005 as in 2000. By 2010, however, the highest stress area was no longer in the southwestern part of the county near Malmö. It had shifted to the northwestern part of the county.

### Sociodemographic and commuting characteristics in different areas

Sociodemographic and commuting characteristics of participants in the highest and lowest stress regions were compared to the county as a whole at baseline and follow-up (Tables 1, 2, 3). Sociodemographic characteristics of 30–60 min car commuters in the entire county varied over time. As expected given the aging of the study subjects over the repeated cross-sections, there was a difference in the proportion of commuters over the age of 50, with the lowest proportion in 2000 and the highest proportion in 2010. The educational status was lowest in 2000 and highest in 2010. There was also some difference in neighborhood connection which was lowest in 2000 and highest in 2010. Income was also highest in 2010 with 8 times the base amount and lowest in 2000 with 6.7 times the base amount.

**Table 1:** Socioeconomic and commuting characteristics of 30–60 min car commuters in highest and lowest stress areas in 2000

	Highest stress area	Lowest stress area	County
Number	N = 68	N = 71	N = 616
Proportion with stress (%)	37** (z = 2.70)	14 (z = 1.54)	22
Female (%)	40 (z = 0.11)	39 (z = 0.17)	41
50 years or older (%)	37 (z = 0.04)	44 (z = 0.90)	38
More than 12 years of school (%)	50 (z = 0.00)	47 (z = 0.42)	50
White collar workers (%)	67 (z = 0.39)	71 (z = 1.16)	64
Employed full time (%)	90 (z = 0.37)	90 (z = 0.48)	88
High job satisfaction (%)	82 (z = 1.50)	68 (z = 0.93)	73
High neighbourhood connection (%)	39 (z = 0.09)	38 (z = 0.22)	40
Living alone (%)	9 (z = 0.00)	6 (z = 0.89)	10
Median income	7.3	6.4	6.7
Greater than median income (%)	53 (z = 0.37)	44 (z = 0.84)	Ref
Number fulfilling distance criteria	N = 61 (470)	N = 58 (473)	N = 531
Working in Malmö/Lund (%)	61** (z = 1.97)	28** (z = 2.87)	47
Living in Malmö/Lund (%)	7** (z = 3.89)	0** (z = 4.88)	30
Work or live in Malmö/Lund (%)	62 (z = 0.91)	28** (z = 4.18)	56
Commuting distance over median (%)	59 (z = 1.28)	55 (z = 0.66)	Ref
Mean commuting distance (km)	28	29	26
Median commuting distance (km)	27	27	24

Italics text and ** highlight statistically significant values at the 95 % level (p < 0.05) and italics text and * highlight statistically significant values at the 90 % level (p < 0.10) in comparison to the rest of the county for 2000

**Table 2:** Socioeconomic and commuting characteristics of 30–60 min car commuters in highest and lowest stress areas in 2005

	Highest stress area	Lowest stress area	County
Number	N = 38	N = 31	N = 543
Proportion with stress (%)	24 (z = 0.60)	10 (z = 1.04)	19
Female (%)	29* (z = 1.65)	61* (z = 1.82)	43
50 years or older (%)	42 (z = 0.66)	64 (z = 1.57)	49
More than 12 years of school (%)	63 (z = 0.62)	64 (z = 0.67)	57
White collar workers (%)	68 (z = 0.27)	79 (z = 1.48)	65
Employed full time (%)	95 (z = 1.63)	90 (z = 0.76)	84
High job satisfaction (%)	78 (z = 0.00)	84 (z = 0.63)	78
High neighbourhood connection (%)	45 (z = 0.20)	58 (z = 0.99)	48
Living alone (%)	0 (z = 1.56)	6 (z = 0.04)	8
Median income	8.2	7.1	7.4
Greater than median income (%)	63 (z = 1.50)	48 (z = 0.00)	Ref
Number fulfilling distance criteria	N = 33 (436)	N = 26 (443)	N = 469
Working in Malmö/Lund (%)	73** (z = 2.75)	15** (z = 3.06)	47
Living in Malmö/Lund (%)	0** (z = 3.50)	0** (z = 3.06)	29
Work or live in Malmö/Lund (%)	72.7* (z = 1.85)	15** (z = 3.88)	55
Commuting distance over median (%)	54 (z = 0.35)	54 (z = 0.20)	Ref
Mean commuting distance (km)	28	29	27
Median commuting distance (km)	26	27	25

Italics text and ** highlight statistically significant values at the 95 % level (p < 0.05) and Italics text and * highlight statistically significant values at the 90 % level (p < 0.10) in comparison to the rest of the county for 2005

**Table 3:** Socioeconomic and commuting characteristics of 30–60 min car commuters in highest and lowest stress areas in 2010

	Highest stress area	Lowest stress area	County
Number	N = 106	N = 34	N = 454
Proportion with stress (%)	24* (z = 1.90)	9 (z = 0.95)	16
Female (%)	43 (z = 0.32)	62* (z = 1.75)	45
50 years or older (%)	51 (z = 0.03)	59 (z = 0.68)	51
More than 12 years of school (%)	50 (z = 1.59)	58 (z = 0.00)	59
White collar workers (%)	57* (z = 1.74)	53 (z = 1.40)	66
Employed full time (%)	89 (z = 1.16)	71* (z = 1.92)	84
High job satisfaction (%)	71 (z = 0.28)	76 (z = 0.17)	73
High neighbourhood connection (%)	52 (z = 0.00)	38 (z = 1.40)	52
Living alone (%)	8 (z = 0.23)	12 (0.48)	9
Median income	7.6	7.7	8
Greater than median income (%)	41* (z = 1.80)	47 (z = 0.14)	Ref
Number fulfilling distance criteria	N = 93 (320)	N = 30 (383)	413
Working in Malmö/Lund (%)	29** (z = 3.51)	7** (z = 4.31)	48
Living in Malmö/Lund (%)	0** (z = 5.55)	0** (z = 3.01)	26
Work or live in Malmö/Lund (%)	29** (z = 5.18)	7** (= 5.28)	56
Commuting distance over median (%)	60* (z = 1.88)	30** (z = 2.00)	Ref
Mean commuting distance (km)	31	27	29
Median commuting distance (km)	30	23	28

Italics text and ** highlight statistically significant values at the 95 % level (p < 0.05) and italics text and * highlight statistically significant values at the 90 % level (p < 0.10) in comparison to the rest of the county for 2010

In the areas of high and low stress, few marked differences in the sociodemographic characteristics of 30–60 min car commuters were observed, except in 2010. In 2010, 30–60 min car commuters in the highest stress area were less likely to be white collar workers, and had lower incomes than 30–60 min car commuters in the county as a whole (Table 3). In the lowest stress area, 30–60 min car commuters were less likely to be employed full time and more likely to be female.

In terms of commuting characteristics, commuting distance among 30–60 min car commuters in the study area was higher in 2010 compared to 2000, consistent with trends in Sweden. In each year, the mean and median commuting distances of 30–60 min car commuters in the high stress area were higher than in the county as a whole. The mean and median commuting distances of 30–60 min car commuters in the lowest stress areas were also higher than in the county as a whole, except in 2010 when they were lower.

Except in 2010, commuting by car greater than the median distance for the county was not significantly associated with higher or lower stress. In 2010, the relative distance of the commute and the workplace destination distinguished commuters in the highest and lowest stress areas. In the high stress area, 30–60 min car commuters were more likely to commute over distances exceeding the median distance for the county. A much lower proportion of commuters in the low stress area commuted distances greater than the median for the county as a whole.

Living and working in the Malmö/Lund area had significantly different proportions in the highest and lowest stressed areas in all three years. In 2000 and 2005, commuters in the high stress area were more likely to work in Malmö/Lund and less likely to live in Malmö/Lund, meaning they were commuting from outside into these urban centers. In 2010, however, commuters in the high stress area were less likely to live or work in Malmö/Lund because the high stress area shifted to the northwest.

### Changes in commuting over time

**Fig. 3:**
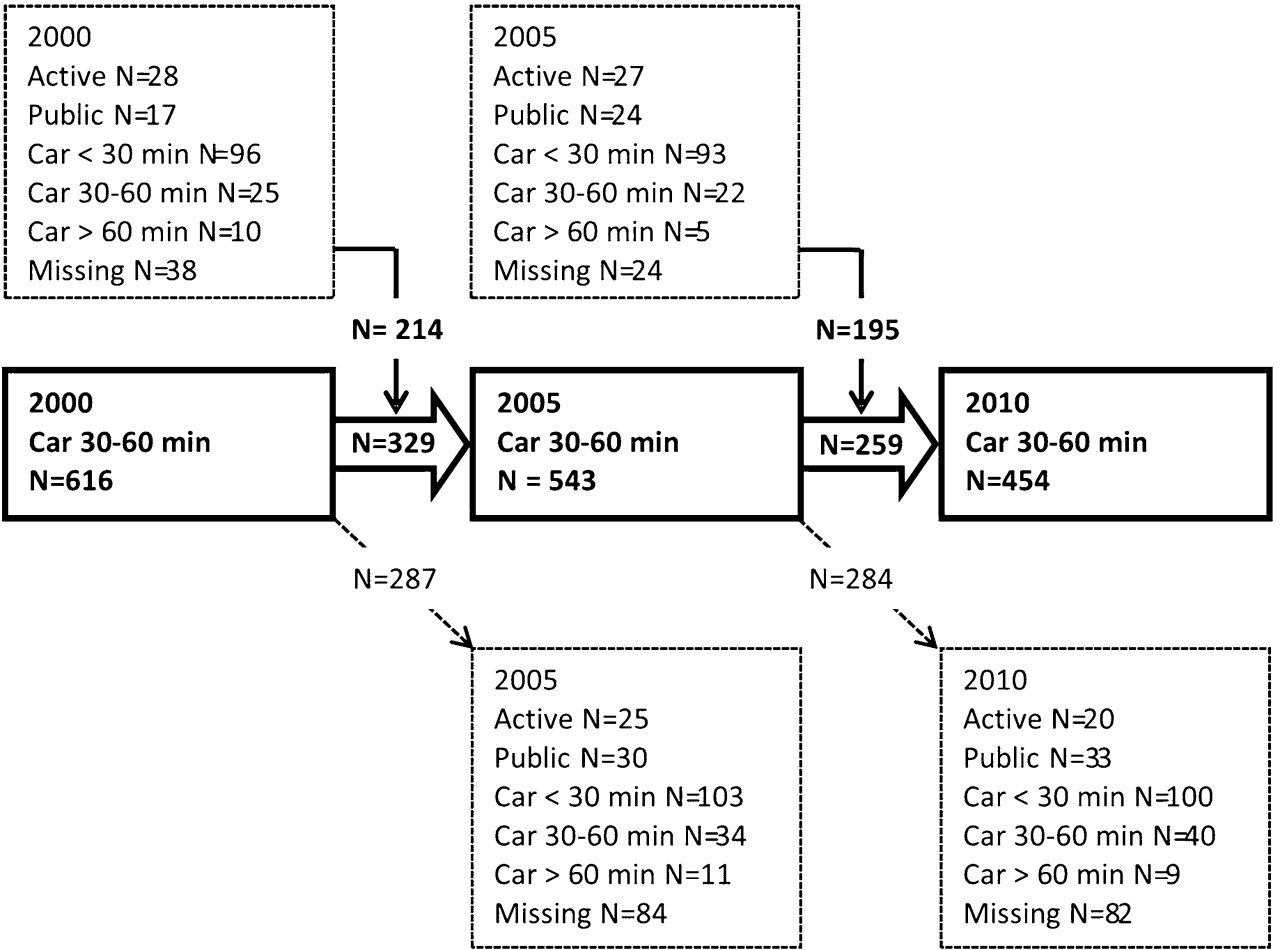
Change in the number of 30–60 min car commuters fulfilling the inclusion criteria at baseline and follow-up. *Bold boxes* show participants who fulfilled the inclusion criteria in a given year and *bold arrows* show participants that also fulfilled the inclusion criteria in the subsequent year. *Dashed boxes* above the *bold boxes* show participants who did not fulfill the inclusion criteria in a given year but fulfilled the inclusion criteria in the subsequent year. *Dashed boxes* below show participants who did fulfill the inclusion criteria in a given year did not fulfill the inclusion criteria in the subsequent year

People change jobs, residential locations, and commuting modes over time. This affects the number of survey participants meeting the inclusion criteria across the years of the study. As noted above, the number of survey participants in the county as a whole who met the inclusion criteria declined from 616 in 2000, to 545 in 2005, and to 454 in 2010. This change is the net result of three processes (Fig. 3). First, from 2000 to 2005, 329 of the survey participants who met inclusion criteria for 2000 also met all of the inclusion criteria in 2005. Second, 287 of the survey participants who met all of the inclusion criteria in 2000 did not meet all of the criteria in 2005 and were excluded in 2005. Third, 214 survey participants who did not meet all of the inclusion criteria in 2000 met the criteria in 2005 and were included in 2005. This is a difference of 73 people, consistent with the net decline in the number of 30–60 min car commuters from 616 in 2000 to 543 in 2005. The corresponding figures for 2005 and 2010 are also shown along with the commuting modes and times of individuals who did not meet the inclusion criteria in a given year.

From 2000 to 2005 and from 2005 to 2010, more than half the 30–60 min car commuters included in the study maintained a 30–60 min car commute. From 2000 to 2005 and from 2005 to 2010, the most frequently observed commuting mode and duration among those survey participants who did not meet the inclusion criteria in every year of the study was a commute by car of <30 min. The results for the county as a whole indicate that many car commuters changed their patterns of commuting over the study period resulting in increases or decreases in reported commuting time.

Among the 68 commuters in the highest stress area in 2000, only 56 % of those reporting high stress in 2000 had the same residence by 2010 and 72 % had a different workplace by 2010 (Table 4). Two-thirds of those reporting high stress in 2000 were still commuting 30–60 min by car in 2010. Among the 30–60 min commuters reporting low stress in 2000, 50 % had changed to a shorter car commute or some other mode by 2010.

**Table 4:** Changes in residence, workplace, commuting and stress level from 2000 to 2010 among residents of the 2000 highest stress area

	Residents reporting high stress in 2000 (N = 25) (%)	Residents reporting low stress in 2000 (N = 43) (%)
Residence in 2010		
Same as 2000	56	49
Different inside area	24	30
Different outside area	20	21
Workplace 2010		
Different from 2000	72	69
Commuting in 2010		
Car <30 min	17	27
Car 30–60 min	67	50
Car >60 min	6	3
Public transit	11	13
Active	0	7
Stress level in 2010		
High	12	9

Of all 30–60 min car commuters living in the highest stress area in 2000 who moved out of the area entirely by 2010, 36 % reported high general stress in 2000. Of individuals who moved within the 2000 high stress area between 2000 and 2010 and commuted 30–60 min by car in 2010, the 2010 stress level was only 18 %. More study subjects moved within the area between 2000 and 2010 than out of the area.

Among the 106 commuters in the highest stress area in 2010 (Table 5), only 8 % of those commuters reporting high stress in 2010 had lived outside the area in 2000, but 86 % had a different workplace location from the one reported for 2000. All of them had commuted by car in 2000, but 30 % had commuted <30 min.

**Table 5:** Changes in residence, workplace, commuting and stress level from 2000 to 2010 among residents of the 2010 highest stress area

	Residents reporting high stress in 2010 (N = 25) (%)	Residents reporting low stress in 2010 (N = 81) (%)
Residence in 2000		
Same as 2010	72	62
Different inside area	20	21
Different outside area	8	17
Workplace 2000		
Different from 2010	86	75
Commuting in 2000		
Car <30 min	30	30
Car 30–60 min	70	51
Car >60 min	0	5
Public transit	0	3
Active	0	11
Stress level in 2000		
High	36	17

Of all 30–60 min car commuters living in the highest stress area in 2010 who had lived outside of the area entirely in 2000, 12 % reported high general stress in 2010. Of 30–60 min car commuters who lived in the 2010 high stress area in 2000 and had moved within the area by 2010, 25 % reported high stress. More of the study subjects moved within the 2010 highest stress area than into it between 2000 and 2010.

## Discussion

This analysis highlights the challenges of studying associations between stress and ongoing behaviors such as commuting. The level of self-reported general stress among 30–60 min car commuters varied geographically within Scania. In addition, the locations of areas where stress levels were high or low changed over time. The results for the county as a whole indicate that many car commuters changed their patterns of commuting over the study period resulting in increases or decreases in reported commuting time. The observed geographical shift in the location of the high stress commuting area over time from the southwest to the northwest could be explained by a number of processes affecting commuting.

Stress levels among 30–60 min car commuters were highest in 2000 and lowest in 2010. This result is also in concordance with the overall stress level in the county based on public health surveys conducted in 2000, 2004, 2008 and 2012. Stress decreased for the county as a whole from 2000 to 2008, followed by an increase in 2012 [42]. The age of the study subjects in the repeated cross-sections used in this research increases with each follow-up which also could explain some of the general decrease in self-reported stress among 30–60 min car commuters from 2000 to 2010. A prior study showed that stress decreased with age [43].

A number of prior studies conducted in other research settings have shown that increasing commuting time is positively associated with stress [5, 8, 44, 45]. Gottholmseder et al. [5] found that an increase of 1 m in commuting duration decreased the probability of feeling relaxed or very relaxed by 0.1 % among Austrian workers. The perception of the commute as being lost time was important. The association between increasing duration of the commute and health outcomes is not always linear. Hansson et al. [6] found the strongest positive association for stress among car commuters who travelled 30–60 min and that stress was less likely among car commuters who travelled shorter or longer times.

Not all studies have found an association between commuting time and stress across different durations of commute. A study of workers at a single company in southern Germany found associations with some health outcomes which were not significant when other variables were controlled, but not with stress [27]. Variability in the associations between commuting duration and the health outcomes studied based on residential location of the participants was not reported. The fact that some research shows adverse health effects from commuting and other research does not is consistent with a central finding of our research that there is spatial variability in the association between commuting and stress within and across study areas.

Local differences in stress among participants were only partly explained by sociodemographic characteristics, suggesting that the context of where the commute takes place is important. Only in 2010 did commuters reporting high levels of stress and residing in the high stress area have occupation, and income characteristics that differed from commuters in the county as a whole. On the other hand, aspects of the commute itself were significant in distinguishing commuters in high stress areas from the 30–60 min car commuters in the county as a whole.

In 2000 and 2005, commuters in the high stress area were much more likely to have commutes to workplaces in Malmö/Lund and workers in the low stress area were much less likely to commute to workplaces in those cities. Conversely, in both the high and the low stress areas, workers were much less likely to live in Malmö/Lund. This suggests that something about the car commuting routes from surrounding areas into Malmö/Lund contributed to higher self-reported stress in these years.

There is a trade-off between commuting and residential location, where pros and cons need to be weighed against each other [3, 46]. The benefits of having a job that is located far from the residence can include higher salary, more prestigious job, lower residential cost and a more attractive housing and amenities [47]. The negative aspects would include having a longer commute, meaning less spare time and more stress. In relation to our results, changing residences and/or changing jobs can be ways of dealing with an undesirable individual commuting situation.

The availability of repeated measures made it possible to study the level of self-reported stress in the same individuals fulfilling the inclusion criteria over time in relation to their commuting patterns. Although many 30–60 min car commuters maintained this pattern of commuting over time, many individuals changed their residential and workplace locations over the study period and others changed their commuting modes. The high level of residential, workplace location and commuting change observed in the high stress area in 2000 suggests that there may be a “healthy commuter” effect [6]. Individuals in commutes that affect health negatively may change their commutes by moving, finding new employment, traveling to work by a different mode, or some combination of these to relieve stress.

In 2010, one out of eight Swedes moved, although people tended to stay close to their prior home and two-thirds moved within the same municipality [48]. Overall the majority of Swedes tend to live in the same place for a long time [49]. Residential relocation almost always affects the distance, time, and route of the commuting trip even if the workplace location and commuting mode remain unchanged.

This, along with spatial heterogeneity in the relationships between commuting and health outcomes, has implications for the design of studies investigating the health effects of commuting. Not everyone has the possibility to change commuting due to income, family situation, or other factors. This could therefore be expected to affect different socioeconomic groups differently, but also different geographical areas due to differences in the possibility to change workplace, residence or mode of commute. A study conducted in northern Sweden showed that the geographical structure of the place shapes commuting. In that study, people living in sparsely populated areas had shorter distances to work, workplaces were concentrated and commuting between them was not considered feasible [49].

Selection for participation in the public health survey was designed to yield a representative status of public health in Scania. The initial sample in 2000 was stratified to represent all 33 municipalities in Scania. The response rate at the baseline was 55 %. A representability analysis showed some underrepresentation in general of men and younger respondents, as well as of persons born outside Sweden [50]. Some selection bias is probably introduced due to the application of the inclusion criteria. The questionnaire was not specifically aimed at commuters and thereby avoids report bias.

The stress measure captured everyday stress levels and not just the stress that could be attributed to commuting. Finding connections between car commuting between 30 and 60 min and stress would therefore suggest a strong influence on the everyday stress for these commuters. Everyday stress was chosen as the outcome in this study as it could be expected to occur in close relationship to the exposure of the commuting. However, other adverse health measures, such as sleep disturbance and low self-rated health have been related to car commuting in the Scanian population [6]. Stress was measured with a one-item scale and the specificity of this measure can be argued, but a similar one-item scale has been used in prior studies [5].

Commuting mode and time were reported retrospectively in 2010 for 2000 and 2005 and some recall bias might be present due to this. Actual travel routes were not known, so Euclidean distance was calculated based on the residence and the workplace location. Euclidean distance has been shown to be a good proxy for travel distances in health studies [51].

The question about commuting mode does not distinguish between drivers and passengers, which is a limitation due to that there can be difference in the experience of stress among car occupants. Morris and Guerra [8] found that long trips were associated with stress among car drivers but not passengers.

The study design is primarily cross-sectional, even though individuals participated in repeated waves. The association between 30 and 60 min car commuting observed in this research, especially strong in particular areas within the county, could mean that this form of commuting contributes to everyday stress. On the other hand, it could also mean that commuters in stressful life situations might choose to commute by car as a response to stress, as this mode is often considered to be the fastest or most flexible [6]. The role of commuting as a cause of stress or other health problems merits additional study.

We were not able to integrate data on levels of congestion or other factors such as construction affecting the car commuting routes in different settings. There are a number of factors that could cause stress from car commuting that are related to the environment and unevenly distributed geographically. These include congestion, noise, air pollution, the experience of the commute, accessibility, and other drivers’ behavior. More research is needed to develop methods for characterizing commuting environments and monitoring levels of stress experienced during and after trips to work. Geographic information systems can plan an important role in integrating these data with other information on individual commuter and trips to work.

Stress has been measured in different ways in studies of the health effects of commuting. In our study perceived everyday stress was used in order to capture stress that could be closely related in time with the commuting exposure. Koslowsky et al. also adopted a measure of perceived stress in studying 200 commuters in Tel Aviv [45]. Gottholmseder et al. used a survey question about how stressed 697 employees in Austria felt when arriving at work, based on a 4-point Likert scale (very stressed, stressed, relaxed, very relaxed) [5]. Research has also relied on multiple measures of stress. Salivary cortisol levels along with perceived stress were used by Evans and Wener to measure stress among 208 suburban railway commuters in New York [52]. In a study of 56 railway commuters in New York, Evans et al. compared the association between cortisol levels and perceived stress (measured on a six-item, five point Likert scale) and found that both measures were positively correlated to unpredictability of the commuting trip but perceived stress was more strongly correlated [53]. Further research, especially using new technologies for collecting real-time data related to stress before, during, and after the commute, would be of value.

In this study, we focused on spatial variability in everyday stress among 30–60 min car commuters. Car commuters with commutes <30 min had similar levels of stress as the group we studied declining from 25 % in 2000 to 19.7 % in 2010. Stress levels among car commuters traveling more than 60 min were more variable across the 3 years in the study. Future research to compare patterns across these groups is a logical next step, provided that the numbers in these groups are sufficiently large. In 2010, there were only 46 individuals commuting more than 60 min by car. Assessing the degree of overlap in high and low level stress areas for different groups of commuters would help to identify problem areas and further improve the understanding the associations between stress and commuting and spatial patterns in these associations.

Very few longitudinal studies exploring the impact of commuting on stress, health and well-being have been conducted and the need for studies with this type of design is great. They have the potential to provider greater insight into how individuals cope with stressful commutes and how individuals with stress arising from other aspects of life choose to commute.

Like all research analysing geographic data, this study of commuting and everyday stress is grounded in place. Nevertheless, our work has implications beyond the specific locale in southern Sweden. As noted, the patterns of commuting observed in Scania in terms of mode and distance are similar to patterns of commuting in the other major metropolitan regions of Sweden, Europe, and North America [3, 25, 26]. There is a broad interest in studying the association between commuting and health in many countries including Germany [3], Austria [5], Sweden [6], US [8], UK [10], Canada [19] and Australia [20]. Our research illustrates a method for investigating whether the association between commuting and stress is the same everywhere. Given the different results across studies conducted in different places, there is a great need to investigate spatial patterns, individual and commuting characteristics which might be associated with them, and changes in these patterns over time. Spatial statistics such as the geographically weighted proportion used in this research are well-suited to uncover the key patterns. The methodology we use can be adapted to other study settings where individual-level data on residential location, commuting mode and time, and health status are available over time.

Our work has important implications for analyzing health data. The associations observed at one geographic scale such as the nation or the county may not be uniform when observed for other geographic scales such as the local community level. The level of stress among commuters in two counties could be the same but the sub-county patterns could be very different. In one county, the level of stress could be almost the same everywhere while in another county, as observed in this research, the level of stress reported by commuters could be higher in some areas. If the association between commuting mode and time and self-reported stress is the same everywhere, there is likely something about the behavior itself that is associated with the undesirable health effect. If spatial variability in the association is observed, other factors may be affecting the association between community mode and time and self-reported stress. These include characteristics of the local population and characteristics of the local environment. Health analysts and policymakers at a national level need to understand patterns of spatial variability at different scales and the factors contributing to variability. As noted, our work is novel in that few studies have investigated spatial variability and few studies have examined this question over time.

## Conclusion

Commuting is an inherently geographic process, involving travel from home to work using different corridors of movement. As an important component of the working day, commuting has implications for health. Our findings confirming spatial non-stationarity support and give context for the apparently contradictory results of previous work on the relationship between commuting and stress. Conducted in different settings, some studies found an association and others did not. Spatial heterogeneity in the relationship between car commuting and stress observed in Scania suggests that spatial analysis of commuting patterns and a range of health conditions is needed to identify the sets of places where the health effects of different modes and duration of commuting are similar.

Health analysts and policymakers at the national level need to understand patterns of spatial variability so that intervention efforts can be directed to those communities where the associations between commuting and health are strongest given the commuting environment. Geographical shifts in the locations of areas where associations with health are strongest highlight the need for health analysts, urban planners, and transportation researchers to collaborate on better ways to characterize the commuting environment. Spatial statistics such as the geographically weighted proportion used in this research are well-suited to uncovering the key patterns. The methodology can be adapted to other study settings where individual-level data on residential location, workplace location, commuting mode and time, and health status are available.

Studies of the relationship between commuting and stress have not generally emphasized change in residence, workplace, or commuting mode over time as possible means of coping. Even less attention has been paid to how health status might affect choice of commuting mode. Longitudinal study designs are needed to capture these dynamic aspects of commuting over time and its connection to the health of workers.

## Abbreviations

PHS: public health survey Public Health in Scania
SCB: Statistics Sweden
GWP: geographically weighted proportions

## References

[1] Mattisson K, Håkansson C, Jakobsson K (2015). Relationships between commuting and social capital among men and women in southern Sweden. Environ Behav.

[2] Hansen KB, Nielsen TAS (2014). Exploring characteristics and motives of long distance commuter cyclists. Transp Policy.

[3] Stutzer A, Frey BS (2008). Stress that doesn’t pay: the commuting paradox*. Scand J Econ.

[4] Koslowsky M, Kluger AN, Reich M (1995). Commuting stress: causes, effects, and methods of coping.

[5] Gottholmseder G, Nowotny K, Pruckner GJ, Theurl E (2009). Stress perception and commuting. Health Econ.

[6] Hansson E, Mattisson K, Bjork J, Ostergren P-O, Jakobsson K (2011). Relationship between commuting and health outcomes in a cross-sectional population survey in southern Sweden. BMC Public Health.

[7] Thoits P (2010). Stress and health: major findings and policy implications. J Health Soc Behav.

[8] Morris EA, Guerra E (2015). Are we there yet? Trip duration and mood during travel. Transp Res Part F Traffic Psychol Behav.

[9] Novaco R, Kliewer W, Broquet A (1991). Home environmental consequences of commute travel impedance. Environ Ecol Psychol.

[10] Gatersleben B, Uzzell D (2007). Affective appraisals of the daily commute: comparing perceptions of drivers, cyclists, walkers, and users of public transport. Environ Behav.

[11] Lin D, Allan A, Cui J (2015). The impacts of urban spatial structure and socio-economic factors on patterns of commuting: a review. Int J Urban Sci.

[12] Feuillet T, Charreire H, Menai M, Salze P, Simon C, Dugas J, Hercberg S, Andreeva VA, Enaux C, Weber C, Oppert JM (2015). Spatial heterogeneity of the relationships between environmental characteristics and active commuting: towards a locally varying social ecological model. Int J Health Geogr.

[13] Helbich M, Böcker L, Dijst M (2014). Geographic heterogeneity in cycling under various weather conditions: evidence from Greater Rotterdam. J Transp Geogr.

[14] Fan JX, Wen M, Kowaleski-Jones L (2014). An ecological analysis of environmental correlates of active commuting in urban US. Health Place.

[15] Axisa JJ, Scott DM, Newbold KB (2012). Factors influencing commute distance: a case study of Toronto’s commuter shed. J Transp Geogr.

[16] Sandow E (2014). Til work do us part: the social fallacy of long-distance commuting. Urban Stud.

[17] Anable J, Gatersleben B (2005). All work and no play? The role of instrumental and affective factors in work and leisure journeys by different travel modes. Transp Res Part A Policy Pract.

[18] LaJeunesse S, Rodriguez DA (2012). Mindfulness, time affluence, and journey-based affect: exploring relationships. Transp Res Part F Traffic Psychol Behav.

[19] Legrain A, El-Geneidy AM, Eluru N (2015). Am stressed, must travel: the relationship between mode choice and commuting stress. Transp Res Part F Traffic Psychol Behav.

[20] Rissel C, Petrunoff N, Wen LM, Crane M (2014). Travel to work and self-reported stress: findings from a workplace survey in south west Sydney, Australia. J Transp Health.

[21] Wener RE, Evans GW (2011). Comparing stress of car and train commuters. Transp Res Part F.

[22] Scania Regional Council. Tillgänglighets atlas—Geografisk tillgänglihet för Skåne (Accessibility Atlas—geographical accessibility in Scania). Department for regional development. 2009. https://skane.se/upload/Webbplatser/Statistik/Koncept-Tillganglighetsatlas%20070827.pdf. Accessed 03 June 2016.

[23] Swedish Government Offical Reports. Flyttning och pendling i Sverige (Moving and commuting in Sweden). SOU; 2007:35. http://www.regeringen.se/contentassets/4b92473a96d544c68b9dfb4e86cbb013/sou-200735-flyttning-och-pendling-i-sverige. Accessed 03 June 2016.

[24] Wahl C, Ullberg M. Resvaneundersökning 2013 (Travel survey 2013). City of Malmö. 2014. http://malmo.se/download/18.50dab45f146afe8fc2c25f6/1403788909668/RVU2013Sk%C3%A5ne+1.0.pdf. Accessed 03 June 2016.

[25] American Association of State Highway and Transportation Officials. Commuting in America 2013—The national report on commuting patterns and trends. AASHTO. 2013. http://traveltrends.transportation.org/Documents/B1_CIA_Overview_web_2.pdf. Accessed 03 June 2016.

[26] Eurostat. Passenger transport statistics. http://ec.europa.eu/eurostat/statistics-explained/index.php/Passenger_transport_statistic. 2016. Accessed 02 June 2016.

[27] Mauss D, Jarczok MN, Fischer JE. Daily commuting to work is not associated with variables of health. J Occup Med Toxicol. 2016;11(12).doi:10.1186/s12995-016-0103-z**(eCollection 2016)**. 10.1186/s12995-016-0103-z PMC4802927 27006685

[28] Turner JR, Wheaton B, Lloyd DA (1995). The epidemiology of social stress. Am Sociol Rev.

[29] Bak CK, Tanggaard Andersen P, Bacher I, Draghiciu Bancila D (2012). The association between socio-demographic characteristics and perceived stress among residents in a deprived neighbourhood in Denmark. Eur J Pub Health.

[30] Scania Regional Council. Fler kärnighet i Skåne (Multiple cores in Scania). Department for regional development. 2011. http://utveckling.skane.se/publikationer/rapporter-analyser-och-prognoser/flerkarnighet-i-skane/Folkhälsorapport. Accessed 03 June 2016

[31] Weimann H, Rylander L, Albin M, Skärbäck E, Grahn P, Östergren P-O, Björk J (2015). Effects of changing exposure to neighbourhood greenness on general and mental health: a longitudinal study. Health Place.

[32] Clark B, Chatterjee K, Melia S (2015). Changes in level of household car ownership: the role of life events and spatial context. Transportation..

[33] Roberts J, Hodgson R, Dolan P (2011). “It’s driving her mad”: gender differences in the effects of commuting on psychological health. J Health Econ.

[34] Solá GA, Vilhelmson B (2012). Convergence or divergence? Changing gender differences in commuting in two Swedish urban regions. Eur J Geogr.

[35] Dolan P, Peasgood T, White M (2008). Do we really know what makes us happy? A review of the economic literature on the factors associated with subjective well-being. J Econ Psychol.

[36] Burke R (2002). Work stress and women’s health: occupational status effects. J Bus Ethics.

[37] Novaco R, Stokols D, Milanesi L (1990). Objective and subjective dimensions of travel impedance as determinants of commuting stress. Am J Community Psychol.

[38] Ommeren J, Rietveld P (2007). Compensation for commuting in imperfect urban markets. Pap Reg Sci.

[39] Cromley E (2007). Risk factors contributing to motor vehicle collisions in an environment of uncertainty. Stoch Environ Res Risk Assess.

[40] Fotheringham AS, Brunsdon C, Charlton M (2002). Geographically weighted regression: the analysis of spatially varying relationships.

[41] Fleiss JL (1981). Statistical methods for rates and proportions.

[42] Scania Regional Council. Folkhälsorapport Skåne 2013—en undersökning om vuxnas livsvillkor, levnadsvanor och hälsa (Public health report Scania 2013—An investigation of adults living conditions and health). 2013. http://utveckling.skane.se/siteassets/publikationer_dokument/folkhalsorapport_skane_2013.pdf. Accessed 03 June 2016.

[43] Mroczek DK, Almeida DM (2004). The effect of daily stress, personality, and age on daily negative affect. J Personal.

[44] Sposato RG, Röderer K, Cervinka R (2012). The influence of control and related variables on commuting stress. Transp Res Part F Traffic Psychol Behav.

[45] Koslowsky M, Aizer A, Krausz M (1996). Stressor and personal variables in the commuting experience. Int J Manpow.

[46] Green AE, Hogarth T, Shackleton RE (1999). Longer distance commuting as a substitute for migration in Britain: a review of trends, issues and implications. Int J Popul Geogr.

[47] Sandow E, Westin K (2010). The persevering commuter—duration of long-distance commuting. Transp Res Part A Policy Pract.

[48] Statistics Sweden. Inrikes omflyttningar (Domestic movements). http://www.scb.se/Statistik/BE/BE0101/2010A01L/Inrikes_omflyttning.pdf; 2010. Accessed 20 Oct 2015.

[49] Westin K, Sandow E (2010). People’s preferences for commuting in sparsely populated areas: the case of Sweden. J Transp Land Use.

[50] Scania Regional Council. Hälsoförhållanden i Skåne—Folkhälsoenkät Skåne 2000 (Health conditions in Scania—Public health survey Scania 2000). The Unit for Social Medicine. 2000. https://snd.gu.se/sv/catalogue/file/3275. Accessed 03 June 2016.

[51] Jones SG, Ashby AJ, Momin SR, Naidoo A (2010). Spatial implications associated with using Euclidean distance measurements and geographic centroid imputation in health care research. Health Serv Res.

[52] Evans GW, Wener RE (2006). Rail commuting duration and passenger stress. Health Psychol.

[53] Evans GW, Wener RE, Phillips D (2002). The morning rush hour: predictability and commuter stress. Environ Behav.

